# Host-Parasite-Bacteria Triangle: The Microbiome of the Parasitic Weed *Phelipanche aegyptiaca* and Tomato-*Solanum lycopersicum* (Mill.) as a Host

**DOI:** 10.3389/fpls.2017.00269

**Published:** 2017-03-01

**Authors:** Lilach Iasur Kruh, Tamar Lahav, Jacline Abu-Nassar, Guy Achdari, Raghda Salami, Shiri Freilich, Radi Aly

**Affiliations:** ^1^Department of Weed Science, Newe Ya’ar Research Center, Agricultural Research OrganizationRamat Yishay, Israel; ^2^Department of Biotechnology Engineering, ORT Braude CollegeKarmiel, Israel; ^3^The Institute of Plant Sciences, Newe Ya’ar Research Center, Agricultural Research OrganizationRamat Yishay, Israel

**Keywords:** broomrapes, parasitic weed, endophytic bacteria, tomato (*Solanum lycopersicum*), biocontrol

## Abstract

Broomrapes (*Phelipanche/Orobanche* spp.) are holoparasitic plants that subsist on the roots of a variety of agricultural crops, establishing direct connections with the host vascular system. This connection allows for the exchange of various substances and a possible exchange of endophytic microorganisms that inhabit the internal tissues of both plants. To shed some light on bacterial interactions occurring between the parasitic *Phelipanche aegyptiaca* and its host tomato, we characterized the endophytic composition in the parasite during the parasitization process and ascertained if these changes were accompanied by changes to endophytes in the host root. Endophyte communities of the parasitic weed were significantly different from that of the non-parasitized tomato root but no significant differences were observed between the parasite and its host after parasitization, suggesting the occurrence of bacterial exchange between these two plants. Moreover, the *P. aegyptiaca* endophytic community composition showed a clear shift from gram negative to gram-positive bacteria at different developmental stages of the parasite life cycle. To examine possible functions of the endophytic bacteria in both the host and the parasite plants, a number of unique bacterial candidates were isolated and characterized. Results showed that a *Pseudomonas* strain *PhelS10*, originating from the tomato roots, suppressed approximately 80% of *P. aegyptiaca* seed germination and significantly reduced *P. aegyptiaca* parasitism. The information gleaned in the present study regarding the endophytic microbial communities in this unique ecological system of two plants connected by their vascular system, highlights the potential of exploiting alternative environmentally friendly approaches for parasitic weed control.

## Introduction

*Phelipanche aegyptiaca* (broomrapes) is an obligate holoparasite that attacks the roots of almost all economically important crops in the Solanaceae, Fabaceae, Compositae, Brassicaceae, and Umbelliferae plant families (Parker and Riches, 1993; Westwood et al., 2010). This parasitic weed invades host plants by using a highly specialized detection system for strigolactones (hormones secreted by the host roots), whose presence is essential for the germination of parasite seeds (Yoder, 1999; Bouwmeester et al., 2003; Joel et al., 2006; Cardoso et al., 2011). Lacking functional roots and a photosynthetic system, the parasite develops special intrusive organs (haustorium) that directly connect to the vascular system of the host plants (Westwood et al., 2010; Joel et al., 2013). By developing a metabolic sink stronger than that of the host, the parasite channels the flow of water and nutrients from the host, thereby damaging crop (its host plant) development. Following successful attachment to the host root, the adjacent broomrape tissue grows into a bulbous structure called a tubercle (spider stage) from which short root-like organs arise that can form secondary attachments to neighboring host roots. After approximately 4 weeks of growth, a floral meristem is produced (shoot stage), which emerges aboveground to flower and disseminate seeds.

This parasite is the main limiting factor in processing-tomato production in numerous Middle East countries (Dor et al., 2014). A wide variety of parasitic weed control methods have been applied in attempts to control broomrape (Joel et al., 2006; Aly, 2007; Aly et al., 2009; Cochavi et al., 2016), most of which are based on chemical sprays that can be windborne and toxic to non-target plants. Therefore there is a need to find alternative solutions to reduce plant–plant parasitization.

One such alternative is to harness endophytic bacteria that naturally inhabit the internal tissues of most plants (Hallmann, 2001). These bacteria often play important beneficial roles in numerous aspects of their host plant’s biology, including enhanced host growth rate, acceleration of seed germination, increased stress tolerance, and the provision of critical nutrients to the host. Endophytic bacteria may also contribute resistance to their hosts by suppressing pathogens and enhancing the plant’s immune system (Azevedo et al., 2000; Rosenblueth and Martínez-Romero, 2006; Ryan et al., 2008; East, 2013). Furthermore, as recently reviewed by Joel et al. (2013), plant endophytes may influence the interaction of their hosts with parasitic weeds, For example the bacterium *Azospirillum brasilense* inhibits seed germination and radical elongation in the broomrape *P. aegyptiaca*; *Pseudomonas fluorescens* reduces both the quantity and biomass of the broomrape *Orobanche foetida* (Zermane et al., 2007); and the bacterium *Rhizobium* spp. reduces not only seed germination of *O. foetida* but also the number of tubercles on its host’s (chickpea) roots (Hemissi et al., 2013). Despite these reports, to date, there is no published information regarding endophytic bacteria inhabiting the parasitic weed during the establishment of parasitism. Furthermore, the parasite and host connect through their vascular system forming a unique ecological system that potentially enables movement of bacteria from one plant to another, resulting in a host-parasite-endophyte triangle. Therefore, the first step in clarifying the role of these bacteria in the parasitism is to investigate the endophytic communities during the phases of parasitization and compared them with parasitized and non- parasitized host tissues.

To do so, we used tomato (*Solanum lycopersicum*) as a host and *P. aegyptiaca* as the parasitic weed.

## Materials and Methods

### Plant Materials

The parasitic weed and its host were grown as was previously described (Eizenberg et al., 2004): *Solanum lycopersicum* L. ‘MP-1’ plants were served as hosts for *P. aegyptiaca* parasitization. The parasitic seeds were collected from an infested tomato field near Qiryat Shemona (northern Israel), dried and kept at 8°C until use. The host plant was planted into 4 L pots filled with soil (light-medium clay with 63% sand, 12% silt, and 22% clay) and grown in a greenhouse under natural lighting with an average 14 h of daylight and a temperature of 20 ± 6°C. These plants were watered and fertilized as needed. Four developmental stages of *P. aegyptiaca* were sampled: seeds, pre-haustorium stage, tubercle (spider stage) and shoot. To ensure that only endophytic bacteria from the plant tissue were being examined, samples were surface-sterilized twice by 2-min incubation in 70% ethanol and 10 min in 6% sodium hypochlorite, followed by a double rinsing in sterile double-distilled water (DDW) (10 min each). To deprive contamination by external bacteria, 100 μl of DDW from the second wash was plated on nutrient agar by Drigalski spatula. In addition, we examined the plant surface by general bacterial probe using FISH analysis (Supplementary Figure S1). No external contamination was detected.

### Characterization of Community Composition of Associated Endophytic Bacteria in *P. aegyptiaca* and its Host Root

DNA was extracted from plant tissue using the cetyltrimethylammonium bromide (CTAB) method (Chen and Ronald, 1999).

DNA from sterilized tissues of non-parasitized tomato roots, parasitized tomato roots (collected a few cm from parasite-attachment point) and from the tubercle of the parasite (spider stage). PCR was performed using general 16S rRNA bacterial primers, which reduce plastid amplification (63F+1401R) (Supplementary Table S1). The PCR products were sent for sequencing to the DNA Services Facility (Chicago, IL, USA) and 10 μl of each reaction was kept at −20°C as a reference. Sequencing was performed by high-throughput amplicon sequencing using the Illumina MiSeq platform at Research and Testing Laboratory (Lubbock, TX, USA) with the primers 515F+806R (Supplementary Table S1). We used five replicates for each treatment.

### Characterization of Community Composition of Associated Microbes of *P. aegyptiaca* (the Parasitic Weed) Across its Developmental Stages

To identify the bacterial community associated with *P. aegyptiaca*, DNA was extracted at different stages of the parasite development (pre-haustorium, tubercles and shoots) and PCR analysis was performed using general 16S rRNA bacterial primers, which reduce plastid amplification (27F+783R). PCR products were isolated with AgencountAMPure^®^ XP beads (Beckman Coulter, Nyon, Switzerland) and next generation sequencing was performed by Ion Torrent^TM^ sequencer (Life Technologies, Grand Island, NY, USA) using primers 27F+338R (Supplementary Table S1). We used three replicates for each treatment.

**FIGURE 1:**
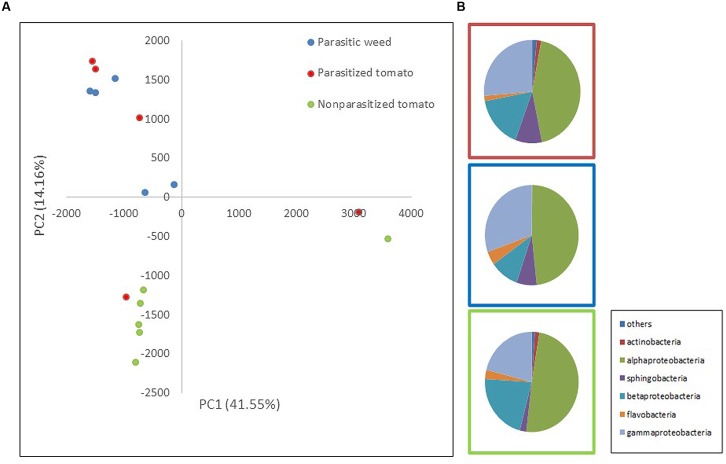
**Comparison of endophytic communities based on mass sequencing of ribosomal 16S rRNA gene.** The bacterial community composition was determined for parasitized tomato root (red), parasite (*Phelipanche aegyptiaca* – spider stage) (blue) and non-parasitized tomato root (green) in five repeats each. Sequences were classified into operational taxonomic units using a 97% similarity threshold. **(A)** The first and second dimensions of PCA analysis. **(B)** Relative abundance of bacterial classes among treatments (an average of all five repeats).

**FIGURE 2:**
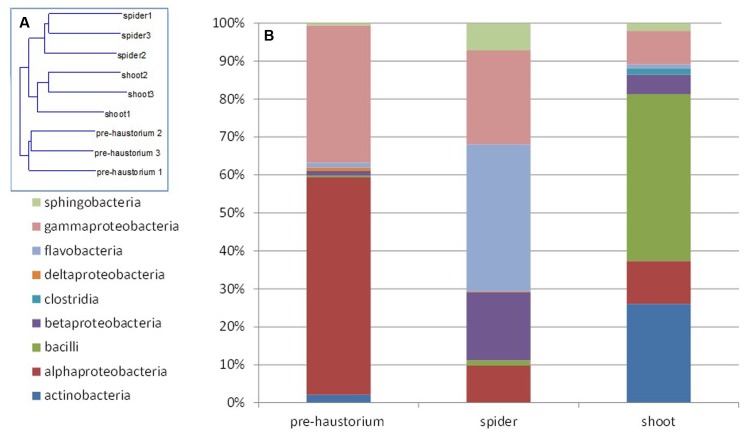
**Endophytic community composition at different stages of the parasite development as obtained from 16S rRNA gene mass sequencing.** The bacterial community composition was determined for *P. aegyptiaca* germinated seeds before attachment to the host root (pre-haustorium), *P. aegyptiaca* tubercles (spider stage) and shoot, after attachment to the host root. Sequences were classified into operational taxonomic units using a 97% similarity threshold. **(A)** Neighbor-joining tree calculated by Rho. **(B)** Relative abundance of bacterial class at different parasite developmental stages.

Two mass-sequencing methods and primer sets were applied to the parasitic weeds at the spider stage and the resultant community composition data were similar, indicating that both can be used interchangeably. Still, we treated these two dataset independently (**Figures 1**, **2**).

### Microbial Community Analysis

Retrieved sequences were analyzed using MOTHUR software (Schloss et al., 2009). Sequences shorter than 150 bp, as well as those of low quality (multiple N, chimeras, etc.), were omitted. Bacterial sequences were aligned using the Silva-compatible alignment database and a distance matrix was calculated. Sequences were grouped into OTUs at a 97% sequence similarity threshold (i.e., sequences that differed by 3% were clustered in the same OTU). A “sub.sample” command was performed and all samples were adjusted, by random selection, to the sample with the lowest number of sequences (13000 and 1100 sequences per sample for Illumina and ion torrent data, respectively). “Indicator” command was performed in order to identify the specific OTUs (indicator species) of each treatment. Diversity indices, principal component analysis (PCA) and ANOVA statistics (Bray–Curtis distance measure) were calculated using PAST software (Hammer et al., 2001). Diversity indices (Supplementary Tables S2, S3) showing observed OTUs, Chao-1 index of richness – representing the predicted number of OTUs in each sample, Dominance – representing relative abundance distribution among species within each sample and Shannon index of diversity.

### Endophyte Isolation and Identification

In order to make sure that the bacteria in the current study are endophytes, we sterilized the examined plant tissue twice as specified above. Surface-sterilized tissue (100 mg) was homogenized with 1 ml of sterile saline solution (9% NaCl) by sterilized pestle. Lysate (100 μL) was spread on NA plates and kept at 28°C in the dark. Three days after incubation, bacterial isolates were counted and classified according to different colony morphologies. Five repeats were carried out for each treatment.

To determine the phylogenetic affiliation of the bacterial isolates, single colonies were collected and subjected to PCR analysis using 27F+1513R primers targeting the 16S rRNA gene by direct colony PCR method as was previously described (Iasur-Kruh et al., 2011) with primers 27F and 1513R (Supplementary Table S1). The PCR procedure was as follows: DNA was denatured at 95°C for 5 min, followed by 30 cycles at 95°C for 30 s each, 57°C for 30 s and 72°C for 1 min, followed by 5 min at 72°C. The PCR product was sequenced by 3130xl Genetic Analyzer (Applied Biosystems). Phylogenetic affiliation of the isolates was determined by comparing with sequences obtained from the NCBI GenBank database. These strains are kept in glycerol at −20°C in our laboratory collection.

### *In vitro* Seed Germination

Parasite seeds were surface-sterilized, dispersed on Whatman GF/A glass-fiber filters (0.7 cm diameter), covered with the same filter and placed on a Petri dish. After 1 week GR24 (1 mg L^−1^) was added to the disks. To examine the effect of different isolates on the germination, each isolate was grown in LB (Luria broth- Life Technologies, Israel) overnight at 28°C, centrifuged, the pellet was washed with DDW and the culture was adapted to an optical density at 600 nm (OD_600_) of 0.3. The culture was added to the disks with the parasite seeds. After incubation in the dark at 26°C for 7 days, the seeds were rated for seed germination in comparison to seed germination control (without bacteria). The experiment was conducted in five repeats for each isolate.

### *In planta* Test

We used an *in planta* system in which tomato plants were transplanted into polyethylene bags and kept moist with half-strength nutrient solution (Hoagland and Arnon, 1950). Plant growth conditions were 25°C, with 14 h light at 100 μE/s m^2^. Surface-sterilized *P. aegyptiaca* seeds were applied on and around the host roots with care taken to achieve even inoculation of all plant roots. After allowing 7 days for parasite seed preconditioning, 10 mL of 2 mg L^-1^ GR24, a germination stimulant (Mangnus et al., 1992), was added to each bag to synchronize the germination of the *P. aegyptiaca* seeds. To examine the effect of chosen isolates on parasite development, it was grown in LB overnight at 28°C, centrifuged, the pellet was washed with DDW and the culture was adapted to an OD_600_ of 0.4 (equivalent to 1.00E+08 cfu∖ml). The culture was added to the polyethylene bags. After 2–3 weeks, parasitism was evaluated by counting the number of live and dead tubercles on each plant using a binocular microscope. The experiment was conducted in five repeats for each treatment (control and endophytic isolate).

### Sequence Accessions

Sequences obtained from mass sequencing were deposited in the European Nucleotide Archive (ENA) (study accession number: PRJEB7137). Sequences from isolated endophytes were deposited in GenBank (NCBI) (KP219403-KP219415).

## Results

To assess whether bacterial community composition changes following attachment of the parasite (*P. aegyptiaca*) to its host (tomato), we compared the endophytic communities in parasitized and non-parasitized host roots with those found in the parasite during ongoing parasitization (i.e., occurring during the spider stage, the stage of attachment to the host). The results showed that endophytic communities of the parasite and the non-parasitized host differed significantly (*t*-test; *p* = 0.0048). Furthermore, the overall community of the parasitized host root did not differ from that of the parasite on the one hand or from that of the non-parasitized host root (*t*-test; *p* > 0.05) (**Figure 1A**) on the other. A greater number of sphingobacterial sequences were found in the parasitized tomato roots and in the parasite than in the non-parasitized tomato roots (*t*-test; *p* = 0.03) (**Figure 1B**). We observed higher numbers of beta-Proteobacteria and fewer gamma-Proteobacteria in the tomato roots (both non-parasitized and parasitized tomato) in comparison to endophytic community structure of the parasitic weed. Furthermore, there was an introduction of additional taxonomic groups such as actinomycetes in the parasitic weed.

The most abundant OTUs, belonging to the proteobacteria genera; *Rhizobium, Pseudomonas, Comamonadaceae* sp.*, Sphingomonas* and *Burkholderia*, are similarly represented in all three “treatments” (parasitized and non-parasitized tomato roots and parasitic weed) with no significant differences between them. However, examination of specific indicator genera showed that *Novosphingobium* and *Methylophilus* (representing 1 and 0.8% of the total sequence dataset, respectively) were specific for the parasitic weed and *Devosia* (representing 0.5% of the total sequence dataset) was significantly associated with the parasitized tomato root.

Next, we characterized the dynamics of the endophyte communities in the parasitic weed *across* its life cycle, that is, before and after attachment to the host. FISH analysis clearly showed that endophytic bacteria inhabit the inner tissue of all developmental stages of the parasitic weed (Supplementary Figure S1). The endophyte communities of the two stages of the parasite at the post-attachment stages (spider and shoot) were more similar to each other than to those detected in the parasite prior to attachment to the host (pre-haustorium stage) (**Figure 2A**). In the pre-attachment stage the dominant classes found in the parasite were alpha- and gamma-Proteobacteria (mostly represented by *Sphingomonas* and *Acinetobacter* species), comprising 55 and 35% of the bacterial community composition, respectively (**Figure 2B**). Just after attachment, at the spider stage, there was a decrease in the proportions of these classes and an increase in the fraction of Flavobacteria and beta-Proteobacteria (mostly representative of the *Flavobacterium* and *Methylophilus* genera). An increased community of Bacilli and Actinobacteria (40 and 25%, respectively), mostly representative of *Jeotgalibacillus* and *Propionibacterium*, were found at the shoot stage (**Figure 2B**).

In addition, we isolated bacteria from surface-sterilized host tomato roots and from different surface-sterilized developmental stages of *P. aegyptiaca*. Similarly, to the mass sequencing results the endophytic bacteria isolated from pre-haustorium stage belonged to alpha- and gamma-Proteobacteria genera; *Acinetobacter* and *Sphingobium* as well as *Roseomonas* and *Pseudomonas*. Gram positive bacteria were isolated from both the parasite tubercle (spider stage) and its host belonging to *Agrococcus* and *Bacillus* as well as *Roseomonas* and *Pseudomonas* which are gram negative. Unique isolates were identified for the parasite: *Bacillus* sp. and *Rhizobium* sp. and for its host: *Pseudomonas* sp. The diversity of the isolated endophytes was reduced, containing only one *Bacillus* species in the shoot stage and in its host (**Table 1**).

**Table 1:** Phylogenetic identification of bacteria isolated from internal tissues of different developmental stages (pre-haustorium, spider and shoot) of *Phelipanche aegyptica* and from host (tomato roots).

Isolates origin	Isolate name	Isolate closest match in NCBI (accession No. – % seq Identity)	Biological test – seed germination as effected by isolated endophytes
Pre-haustorium	*Acinetobacter* sp. *PhelS2*	*Acinetobacter johnsonii* (KP236314 – 99%)	0%
Pre-haustorium	*Sphingobium* sp. *PhelPH4*	*Sphingobium yanoikuyae* (KC355325 – 99%)	0%
Pre-haustorium	*Roseomonas* sp. *PhelPH5*	*Roseomonas musae* (NR_113233 – 98%)	0%
Pre-haustorium	*Pseudomonas* sp. *PhelS6*	*Pseudomonas stutzeri* (KU749990 – 99%)	0%
Spider and host	*Pseudomonas* sp. *PhelPH1*	*Pseudomonas rhizosphaerae* (KT825699 – 99%)	0%
Spider and host	*Agrococcus* sp. *PhelS3*	*Agrococcus jenensis* (EF672044 – 99%)	0%
Spider and host	*Chryseobacterium PhelS7*	*Chryseobacterium profundimaris* (NR_136427 – 99%)	22%
Spider and host	*Bacillus* sp. *PhelS6*	*Bacillus megaterium* (KU145691 – 99%)	42%
Spider	*Bacillus* sp. *PhelSs*	*Bacillus subtilis* (KX268132 – 99%)	20%
Spider	*Rhizobium* sp. *PhelS9*	*Rhizobium rhizoryzae* (NR_133844 – 99%)	0%
Host	*Pseudomonas* sp. *PhelS10*	*Pseudomonas aeruginosa* (KY203649 – 98%)	80%
Shoot and host	*Bacillus* sp. *PhelSh11*	*Bacillus oceanisediminis* (KX767124 – 99%)	70%

*The effect of these isolates on P. aegyptica seeds in the presence of germination stimulant (GR24) was examined in comparison to control seed (without inoculation of bacteria): 0%- no effect- the same germination as control, 100%- no seed germination observed.*

**FIGURE 3:**
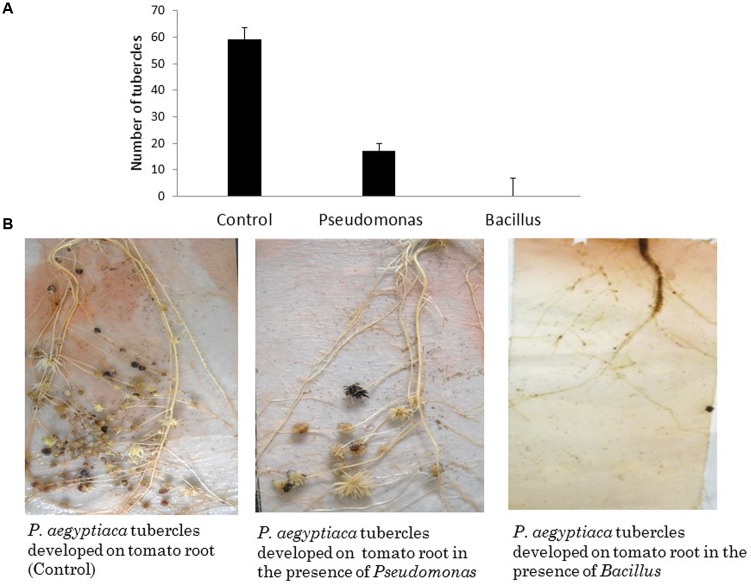
**The effect of *in planta* test of two isolated endophytes (approximately 1.00E+08 cfu∖ml each) on parasitic weed development. (A)** The number of *P. aegyptica* tubercles developing in the presence of selected isolates. **(B)** The effects of the bacterial isolates on *P. aegyptica* tubercles and on tomato roots in PE system. While *Pseudomonas PhelS10* reduced the number of tubercles without harming the host root, the *Bacillus* sp. *PhelSh11* destroyed the host root making the parasitic weed unable to develop. The results are an average of five repeats.

To examine the effect of different endophytic isolates on the interactions between the parasitic weed and its host, we then examined the effect of the isolates on development of the parasitic weed. We screened for the ability of parasite’s seeds to germinate *in vitro* in the presence of each isolate (**Table 1**) and found that two isolates (from the host) reduced the germination of the seeds. *Pseudomonas PhelS10* reduced germination by 80% and *Bacillus* sp. *PhelSh11* reduced it by 70%. These bacteria were then chosen for examination *in planta* using the polyethylene bag test (**Figure 3**). This test showed that the *Bacillus* strain dramatically reduced the viability of tomato roots coincident with the reduction of the parasite tubercles, while the *Pseudomonas* strain reduced the number of tubercles without harming the tomato roots (**Figure 3**).

## Discussion

Endophytes are microorganisms (bacteria, fungi, etc.) inhabiting the inner tissue of plants without causing apparent disease. Tomato is hosting both endophytic fungi and endophytic bacteria, some of them showing the ability to promote seed germination and protect tomato plants from pathogens (Larran et al., 2001; Pozo et al., 2002; Shittu et al., 2009; Xu et al., 2014). Furthermore, since endophytic mycorrhizal fungi contain a large amount of bacteria in the ectomycorrhizal root tips (Sbrana et al., 2002), it might spread endophytic bacteria while colonizing the tomato. de Vega et al. (2010) demonstrated tripartite association among a holoparasitic weed (*Cytinus*), its host Cistaceae species, and mycorrhizal fungi. However, studies on endophytic bacteria in parasitic weed have not been reported yet. In the current work we show that attachment of a parasitic weed to its host has an effect on the bacterial endophytic community composition of both plants. Parasitic weeds are considered ecosystem engineers, due to their ability to induce changes in the abundance and diversity of plants. Such changes may lead to up-stream effects on herbivores, pollinators and seed vectors (Press and Phoenix, 2005). Therefore changes in parasite endophytic communities may affect parasitism and as a consequence plant community makeup. In the current study, we showed for the first time, that bacterial endophytic communities changed with different stages of parasitism, and that they also affect the host’s endophytic composition. The communities inhabiting the root of the host were examined in parallel to the parasite tubercle because this is the stage that the parasite is well-established on the host and the endophytic exchange may occur.

Endophytic bacteria are located *in planta*, where they are protected from the outside environment on the one hand, but are easily affected by changes occurring in the plant tissue on the other (Ryan et al., 2008). The fact that both the parasitic weed and its host endophytic communities changed following parasitization interaction and become more similar to each other can be explained by the following: the parasite affects the environment of the inner tissue of the host plant. Such changes may be exemplified by the reported changes in metabolic profile of the tomato host that occur following parasitization (Hacham et al., 2016). Thus, the changes in endophytic community occurring in the host plant may be affected by the parasite in an indirect manner.

Alternatively, the endophytic community composition is affected in direct manner by an exchange of endophytic bacteria between the *P. aegyptiaca* and its host. Indeed, in the present study the endophytic community of the parasitic weed did not differ from that of the parasitized tomato host but was significantly different from the non-parasitized host. However, the fact that bacterial community composition in the parasitized tomato root is not significantly different from either the non-parasitized tomato root or the parasitic weed itself, makes it difficult to determine the direction(s) in which the bacteria are being transferred. In general endophytic bacterial movement can be either apolplasitc via intercellular space (Maheshwari et al., 2013) or may be facilitated through xylem tubes (Rosenblueth and Martínez-Romero, 2006; Compant et al., 2010). Since the *P. aegyptiaca* tubercle is composed mostly of parenchyma cells which are traversed by both xylem and phloem (Joel et al., 2013), both movement types can serve as potential transmission routes between the parasitic weed and its host through the haustorium bridge.

To date, most of the data in the literature on endophytes and their biological impact on their host plants were established by classical microbiological methods resulting in the loss of much valuable information regarding uncultured endophytes (Hallmann, 2001; Zermane et al., 2007; Hemissi et al., 2013). By using molecular tools we were able to show that even though the dominant endophyte taxonomy in the parasite is similar to that in other known plant endophytes (Lodewyckx et al., 2002), the microbial ecology of these endophytic communities in *P. aegyptiaca* is greatly affected by its connection to the host.

The greater similarity between the endophyte communities of the parasitic weed in the different post-attachment stages to each other, than to those detected prior to attachment to the host (pre-haustorium stage) indicate that connection to the host is affecting the endophytic community composition of the parasite as well as the host. Isolation of and further investigation of indicator bacterial species from both the host and parasite may therefore be of interest. Indeed investigating indicator bacteria such as *Novosphingobium* and *Methylophilus*, from the host *P. aegyptiaca* will enable the understanding of their unique interaction with the parasitic weed.

Endophytes may be equipped with a rich arsenal of metabolites involved in defense, as well as in interaction with the plant (Bouwmeester et al., 2003; Brader et al., 2014), supporting their host plant in different aspects. Therefore, we further examined specific endophytic bacteria isolated from the tomato-parasitic weed system and assessed their effect on *P. aegyptiaca* development. In agreement to previous studies (reviewed by Joel et al., 2013), our results showed that two isolates, associated with tomato-plant, reduced the germination of the parasite’s seeds. Similar mechanisms were shown in a number of studies (Azevedo et al., 2000; Eljounaidi et al., 2016), where bacteria obtained from different crops were shown to protect their hosts against pests and diseases. Furthermore, *Pseudomonas* sp. *PhelS10*, which originated from the inner tissue of the host tomato root, was able to suppress both seed germination (*in vitro*) and parasitism (*in vivo*) of *P. aegyptiaca*, suggesting it may be used by the host for protection against parasitic weed attack. This protection may be through secreting different substances that suppress the pest and∖or enhancing the plant’s immune system (ISR), enabling higher resistance against the parasite as was demonstrated by other beneficial endophytes against other pathogens (Berg et al., 2005; Compant et al., 2005; Innerebner et al., 2011). Therefore it may be possible to harness these naturally occurring microbial partners as possible bio-controls agents against parasitization, reducing the need for harmful chemical pesticide agents.

## Conclusion

In this study, we showed that attachment to the host had a major effect on all tested bacterial parameters of the parasitic weed, suggesting an exchange of endophytes between the host and the parasite. Considering the impact of parasitic weeds on agriculture and the difficulty in establishing efficient control methods further research is required to characterize additional endophytic isolates and to fully optimize this mechanism of resistance. Moreover, a deeper understanding of the relationships in the host-parasite-endophyte triangle may lead to new weed control methods and help alleviate weed-related ecological, agricultural, and economic issues.

## Author Contributions

RA conceived, planned and supervised the work. LI designed and performed the experiments and analyzed the data. SF analyzed the data. TL, JA-N, GA, and RS contributed in data production.

## Conflict of Interest Statement

The authors declare that the research was conducted in the absence of any commercial or financial relationships that could be construed as a potential conflict of interest.
