# Supplementary material for: Host-Parasite-Bacteria Triangle: The Microbiome of the Parasitic Weed Phelipanche aegyptiaca and Tomato-Solanum lycopersicum (Mill.) as a Host
Source: Front Plant Sci. 2017 Mar 1;8:269. doi: 10.3389/fpls.2017.00269 (PMC5331046; doi:10.3389/fpls.2017.00269)
Supplement: Supplementary file 1 [file Data_Sheet_1.PDF]

Table S1 | Primers and probes used in the study.

Primer name	Analysis	Sequence	Reference
27F	PCR for colony sequencing and for Ion torrent analysis	AGAGTTTGATCMTGGCTCAG	(Weisburg et al., 1991)
1513R	PCR for colony sequencing	ACGGYTACCTTGTTACGACTT	(Weisburg et al., 1991)
783R	PCR for Ion torrent analysis	CTACCAGGGTATCTAATCCTG	(Sakai et al., 2004)
338R	PCR for Ion torrent analysis	GCTGCCTCCCGTAGGAGT	(Daims et al., 2001)
Eub338	FISH analysis	Cy3- GCTGCCTCCCGTAGGAGT	(Daims et al., 2001
63F	PCR for Illumina analysis	CAGGCCTAACACATGCADAGTC	(Harasawa et al., 2004)
1401R	PCR for Illumina analysis	GGGTCTTGTACACACCG	(Harasawa et al., 2004)
515F	PCR for Illumina analysis	CACGGTCGKCGGCGCCATT	(Dowd et al., 2008)
806R	PCR for Illumina analysis	GGACTACHVGGGTWTCTAAT	(Dowd et al., 2008)

Table S2 | Diversity indices of endophytic bacterial communities, obtained from parasitic weed (*P. aegyptiaca*), parasitized tomato root (R_O) and non-parasitized tomato root. Diversity indices were calculated based on OTU (97% similarity) assignment of sequence reads. Numbers of observed OTUs, Chao-1 index of richness, Dominance and Shannon index of diversity are presented.

Sample name	Obtained from	Number of OTU	Chao-1	Dominance	Shannon index
O910	*P. aegyptiaca*	672	1276	0.03	4.35
O1112	*P. aegyptiaca*	704	1324	0.03	4.35
O34	*P. aegyptiaca*	847	1441	0.017	4.88
O12	*P. aegyptiaca*	632	1215	0.046	3.89

O78	*P. aegyptiaca*	422	952.2	0.208	2.65
O456	*P. aegyptiaca*	701	1431	0.031	4.15
R_Ob910	Parasitized root	665	1156	0.037	4.33
R_Ob78	Parasitized root	333	727.8	0.069	3.39
R_Ob56	Parasitized root	656	1343	0.066	3.99
R_Ob34	Parasitized root	532	916.6	0.038	4.25
R_Ob12	Parasitized root	833	1619	0.028	4.49
Rcont910	Nonparasitized root	810	1673	0.053	3.92
Rcont78	Nonparasitized root	782	1590	0.043	3.98
Rcont456	Nonparasitized root	600	1100	0.04	4.11
Rcont3	Nonparasitized root	708	1544	0.03	4.23
Rcont1	Nonparasitized root	591	1010	0.065	3.98
Rcont2	Nonparasitized root	557	1117	0.055	3.83

Table S3 | Diversity indices of endophytic bacterial communities, obtained from parasitic weed developmental stages: pre-haustorium, spider and shoot. Diversity indices were calculated based on OTU (97% similarity) assignment of sequence reads. Numbers of observed OTUs, Chao-1 index of richness, Dominance and Shannon index of diversity are presented.

**Sample name**	**Number of OTU**	**Chao-1**	**Dominance**	**Shannon index**
pre-haustorium3	256	846	0.03884	4.202
pre-haustorium2	230	582.4	0.04523	4.024
pre-haustorium1	214	698.5	0.06169	3.765
spider1	228	744.8	0.07398	3.778
spider2	172	457.5	0.09507	3.426
spider3	232	868.5	0.1235	3.656
shoot1	198	474.5	0.03066	4.273
shoot2	148	380.4	0.05238	3.892
shoot3	122	311.8	0.2468	2.594

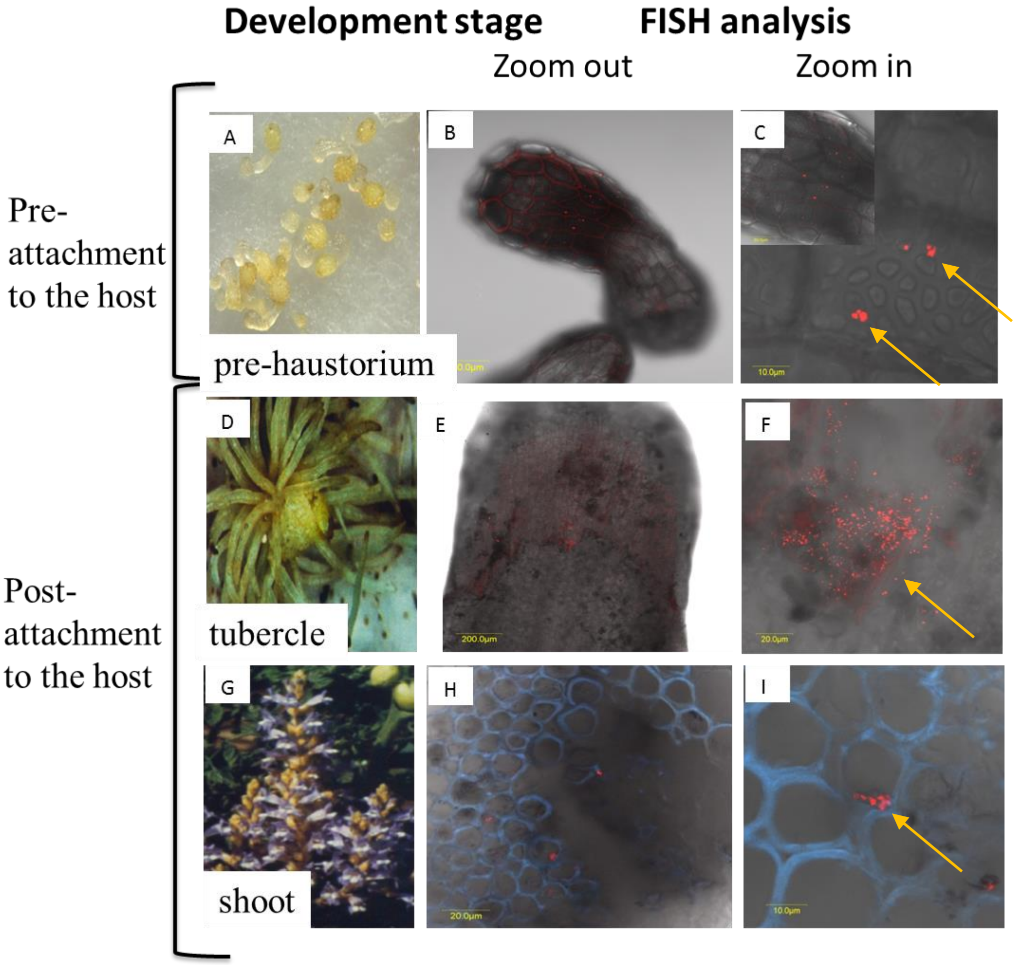

Figure S1 | Fluorescence in situ hybridization (FISH) analysis was used to examine bacteria inside the parasite; *P. aegyptiaca* plants at the three developmental stages were surface sterilized and manually sectioned in triplicate using a razor blade and submerged in FAA (20% ethanol, 2.5% acetic acid, 2.5% formaldehyde, v/v). The samples with FAA were vacuumed for 3 h and left for an additional 20 h at room temperature (RT). Subsequently, they were transferred to 50% ethanol and kept at -20°C until further analysis. The sections were dehydrated by sequential transfer to 70% and 100% ethanol and then submerged in hybridization buffer (20 mM Tris–HCl pH

8.0, 0.9 M NaCl, 0.01% w/v sodium dodecyl sulfate, 30% v/v formamide) with 10 pmol of fluorescent probe (Eub338-(cy3), Table S1) for general bacteria for 4 h at RT. The sections were then washed in PBS and visualized under an IX81 Olympus FluoView500 confocal microscope. Specificity of the detection was confirmed using a no-probe control. The experiment was conducted in triplicates.

Bacterial distribution in *Phelipanche aegyptiaca* at developmental stages pre- and post-attachment to the host after surface sterilization- bacteria were found only in the inner tissue of the plant. Distribution was determine by FISH analysis of tissue sections of the parasite with general probe for bacteria. A, *P. aegyptiaca* germinated seeds (pre-haustorium). B-C, FISH analysis of pre-haustorium with general probe for bacteria. D, *P. aegyptiaca* tubercles (spider stage). E-F, FISH analysis of longitudinal section of the spider stage. G, *P. aegyptiaca* shoot. H-I, FISH analysis of shoot cross-section with general probe for bacteria. No bacteria signal was detected on the surface of the examined tissues. . Red spots and aggregates indicate endophytic bacteria in the parasite cell.
